# AP-1 Recruits SMAP-1/SMAPs to the trans-Golgi Network to Promote Sorting in Polarized Epithelia

**DOI:** 10.3389/fcell.2021.774401

**Published:** 2021-11-25

**Authors:** Shimin Wang, Longfeng Yao, Wenjuan Zhang, Zihang Cheng, Can Hu, Hang Liu, Yanling Yan, Anbing Shi

**Affiliations:** ^1^ Department of Biochemistry and Molecular Biology, School of Basic Medicine, Tongji Medical College, Huazhong University of Science and Technology, Wuhan, China; ^2^ Cell Architecture Research Institute, Huazhong University of Science and Technology, Wuhan, China

**Keywords:** *C. elegans*, polarized sorting, SMAP-1/SMAPs, AP-1, clathrin, intestinal epithelia

## Abstract

Coordinated AP-1 and clathrin coat assembly mediate secretory sorting on the trans-Golgi network (TGN) during conventional secretion. Here we found that SMAP-1/SMAPs deficiency caused the apical protein ERM-1 to accumulate on the basolateral side of the TGN. In contrast, the basolateral protein SLCF-1 appeared abnormally on the apical membrane. SMAP-1 colocalized with AP-1 on the TGN. The integrity of AP-1 is required for the subcellular presence of SMAP-1. Moreover, we found that the loss of SMAP-1 reduced clathrin-positive structures in the cytosol, suggesting that SMAP-1 has a regulatory role in clathrin assembly on the TGN. Functional experiments showed that overexpressing clathrin effectively alleviated exocytic defects due to the lack of SMAP-1, corroborating the role of SMAP-1 in promoting the assembly of clathrin on the TGN. Together, our results suggested that the AP-1 complex regulates the TGN localization of SMAP-1, promoting clathrin assembly to ensure polarized conventional secretion in *C. elegans* intestinal epithelia.

## Introduction

In the conventional secretion pathway, cargo proteins traverse ER-Golgi and reach the plasma membrane via transport vesicles (Mellman and Warren, 2000; Rabouille, 2017; Dimou and Nickel, 2018). There are apical and basolateral membrane domains in epithelial cells, which leads to additional complexity of cargo sorting (Yeaman et al., 1999; Ang et al., 2003; Sato et al., 2007; Nakajo et al., 2016). Accumulating evidence indicated that the trans-Golgi network (TGN) functions as a sorting organelle during secretion in epithelial cells (Mellman and Warren, 2000; Gravotta et al., 2007; Thuenauer et al., 2014). Apical and basolateral proteins must be separated in TGN before their inclusion into separate routes. To ensure polarized secretion, delicate sorting machinery is employed to package the cargo proteins into specific vesicles and then deliver them to various downstream destinations. TGN missorting can lead to inappropriate targeting of cargo proteins and cell polarity defects (Guo et al., 2014).

Cargo adaptors and clathrin are required for the proper TGN sorting. Once recruited onto TGN, cargo adaptors recognize the sorting motif within the cytoplasmic domain of the transmembrane proteins. Then, clathrin is recruited to TGN and thus facilitates sorting the cargos into the specific membrane carriers (Guo et al., 2014). As a heterotetramer, AP complex contains two large subunits (α, β, γ, δ, ε, or ζ), one medium subunit (μ1–μ5) and one small subunit (σ1–σ5) (Nakatsu et al., 2014). Previous studies have shown that AP-1 is implicated in the cargo sorting at the TGN (Brodsky et al., 2001; Nakatsu et al., 2014). In mammals, two AP-1 adaptor complexes have been identified, including AP-1A and AP-1B (Folsch et al., 2003; Shteyn et al., 2011). *C. elegans* genome encodes five AP-1 subunits, including APM-1 (μ1), UNC-101 (μ1), APB-1 (β1), APG-1 (γ), and APS-1 (σ1) (Shim et al., 2000; Zhou et al., 2016). Loss of APM-1 failed to cause uncoordinated (UNC) phenotypes (Shim et al., 2000; Zhou et al., 2016). Instead, UNC-101/AP-1 μ interacts with the bipartite signal within KVS-4/Kv2.1, mediating the polarized sorting of KVS-4 in DA9 neuron (Zhou et al., 2016). Regarding the functionality of clathrin, in addition to clathrin-coated pits during endocytosis, clathrin-coated vesicles also bud from TGN. Arf1 triggers the assembly of the clathrin coat on TGN (Thomas et al., 2021). A mechanistic study revealed that TGN-associated clathrin and AP-1 quickly exchange with free proteins in the cytoplasm, and AP-1 can exchange independently of clathrin (Wu et al., 2003). Together, these results suggested that AP-1 assembly and clathrin assembly are relatively independent events. Additional mechanisms are likely required to couple these two assembly processes, which remains to be elucidated.

Here, we introduced SMAP-1 (stromal membrane-associated protein-1) as a polarized secretion regulator in *C. elegans* intestinal epithelia. SMAP-1 overlapped well with TGN markers, AP-1, and clathrin. Notably, the presence of the AP-1 complex was essential for SMAP-1 localization. Furthermore, we found that SMAP-1 deficiency led to a loss of TGN localization of clathrin. Overexpression of clathrin instead of AP-1 component effectively alleviated secretion defects. In summary, our results suggested that AP-1 governs the TGN localization of SMAP-1, and SMAP-1 acts to facilitate clathrin assembly during polarized secretion.

## Results

### Loss of SMAP-1 Disturbs the Polarized Secretion in the Intestine

**FIGURE 1:**
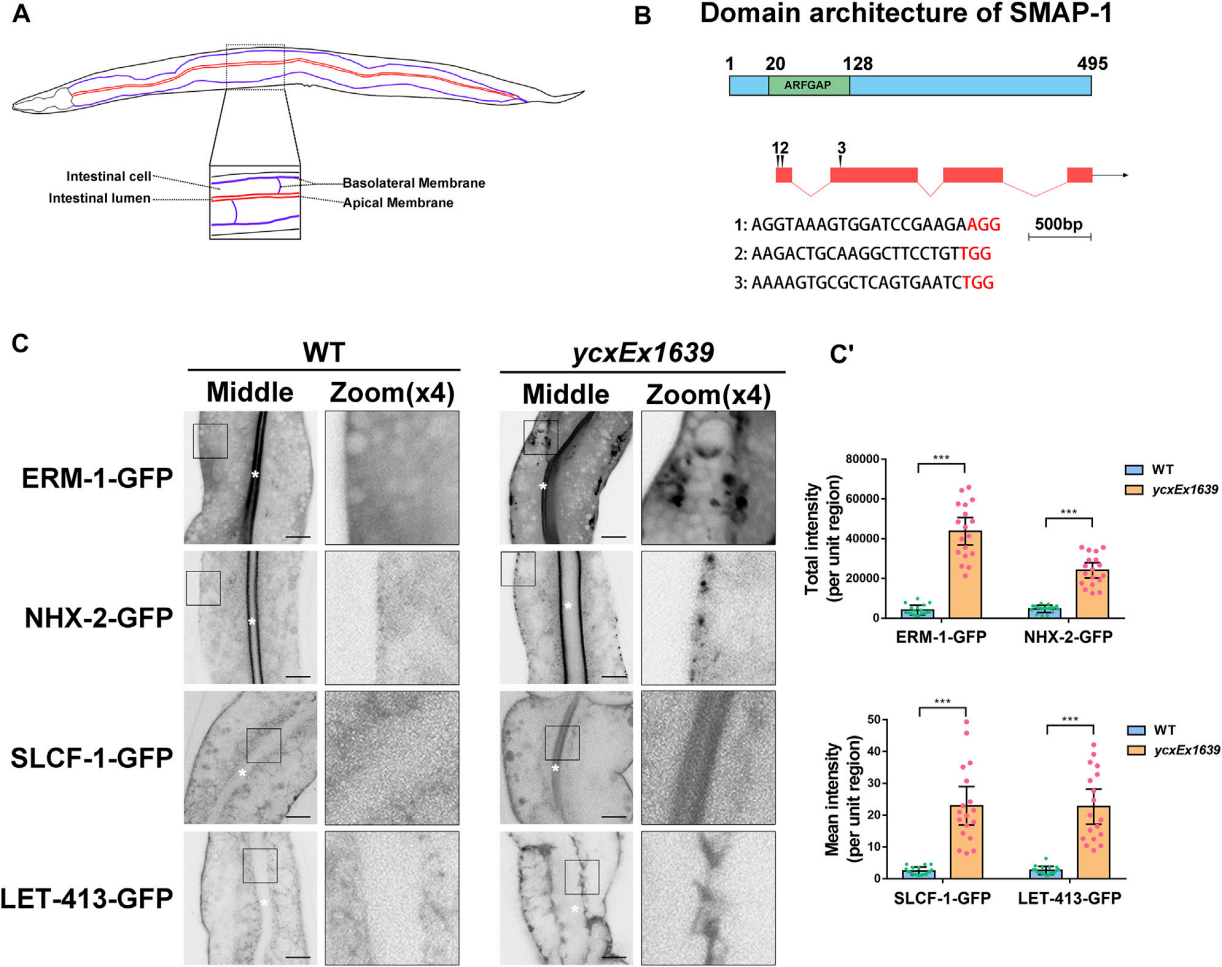
Loss of SMAP-1 disturbs the polarized secretion in the intestine. **(A)** A model of the *C. elegans* intestine indicates the apical and basal sides of intestinal epithelia. **(B)** SMAP-1 contains an N-terminal ArfGAP domain, and amino acid numbers are indicated. **(C-C′)** In *smap-1(ycxEx1639)* mutants, ERM-1-GFP and NHX-2-GFP accumulated on the basolateral side, while SLCF-1-GFP and LET-413-GFP appeared on the apical membrane. White asterisks indicate intestinal lumen. Error bars are 95% CIs (n = 18 each). Asterisks designate the significant differences in the Mann-Whitney test (***p < 0.001). Scale bars, 10 μm.

To better understand the regulatory mechanisms of polarized secretion in epithelia, we deployed apically localized ERM-1-GFP (a membrane-cytoskeleton linker) and basolateral SLCF-1-GFP (a monocarboxylate transporter) as cargos for a genome-wide RNAi screen. We found that loss of W09D10.1 led to defective secretion in the *C. elegans* intestine. *W09D10.1* encodes the sole *C. elegans* homolog of mammalian SMAPs (Funaki et al., 2013; Sato et al., 1998; Tanabe et al., 2005), which we referred to as SMAP-1. Sequence alignment indicated that the N-terminal region (aa 15-119) of SMAP-1 shares 68% identity with SMAP2, while their C-terminal regions lack significant homology. SMAP-1 contains an N-terminal Arf GAP domain (aa 20-128) (Figure 1B). Unlike the C-terminus of SMAP2, which harbors a clathrin-interacting domain and a CALM-interacting domain (Natsume et al., 2006), the C-terminal part of SMAP-1 has not been characterized yet.

In accordance with the predicted expression profile (Spencer et al., 2011), SMAP-1 is broadly expressed in tissues such as the intestine, neuron, and pharynx (Supplementary Figure S1). Whole-animal knockout of SMAP-1 causes larval arrest (Gonczy et al., 2000). Hence, we prepared the transgenic allele *smap-1(ycxEx1639)*, a heat-shock-inducible CRISPR/Cas9 conditional mutant (Supplementary Figure S2A-A′). In *smap-1(ycxEx1639)* intestinal cells (Figures 1A,B), ERM-1-GFP consistently accumulated on the basal side, while SLCF-1 abnormally appeared on the apical membrane (Figure 1C-C′). Similarly, the localization of apical cargo protein NHX-2 (Na+/H+ exchanger) and basolateral recycling regulator LET-413/Erbin were affected (Figure 1C-C′). Previous studies suggested that SMAP2 could act as an Arf1GAP (Arf1 GTPase-activating protein) and regulate the formation of the clathrin coat on the trans-Golgi network (TGN) (Thomas et al., 2021), leading us to examine the distribution of GFP-ARF-1.2. In *smap-1(ycxEx1639)* cells, GFP-ARF-1.2 accumulated in punctate structures (Supplementary Figure S3A-A′), suggesting that SMAP-1 acts as a GAP of ARF-1.2 in intestinal cells. Consistently, overexpression of the SMAP-1 (R60A) variant that lost GAP activity was not sufficient to alleviate cytosolic overaccumulation of ARF-1.2-GFP in *smap-1(ycxEx1639)* cells (Supplementary Figure S3A-A′). The membrane-to-cytosol ratio of ARF-1.2-GFP was increased by ∼70% in *smap-1(RNAi)* animals (Supplementary Figure S3B-B′), and overexpressed SMAP-1-mCherry reduced the puncta labeling of ARF-1.2-GFP (Supplementary Figure S3A-A′).

To verify the role of ARF-1.2 in SMAP-mediated cargo sorting, we examined the localization of ERM-1 and SLCF-1. Notably, the distribution of ERM-1-GFP and SLCF-1-GFP was affected in *arf-1.2(RNAi)* animals (Supplementary Figure S3C-C′). However, ARF-1.2 knockdown failed to alleviate the distributional defects of ERM-1 and SLCF-1 in SMAP-1-deficient cells (Supplementary Figure S3C-C′). Conversely, overexpression of SMAP-1 (R60A)-mCherry rescued the distributional defects of ERM-1 and SLCF-1 (Supplementary Figure S3D-D′). It is noteworthy that Arf1 GTPase has been shown to recruit AP-1 to facilitate the formation of the clathrin coat on the trans-Golgi network (TGN) (Ren et al., 2013; Beacham et al., 2019). Altogether, our results suggested that in addition to acting as a GAP of ARF-1.2 during polarized cargo sorting, SMAP-1 plays an additional role in facilitating sorting in *C. elegans* intestinal epithelia.

ARF-1.2 is required for the retrograde transport from Golgi to the endoplasmic reticulum (ER) (Arakel et al., 2019). To this end, we set to examine the localization of COPB-1 (COP-I complex subunit beta 1), which is expressed in the intestine (Hunt-Newbury et al., 2007; Ackema et al., 2014). As expected, COPB-1 predominantly colocalized with Golgi marker mCherry-P4M (Supplementary Figure S4B-B′). In the absence of SMAP-1, the level of colocalization between COPB-1-GFP and MC-P4M was decreased significantly (Supplementary Figure S4B-B′). Similarly, the Golgi localization of COPG-1 (COP-I complex subunit gamma 1) was reduced (Supplementary Figure S4A-A′). These results indicated that the increase in ARF-1.2 activity induced by SMAP-1 deficiency affected the Golgi recruitment of COP-I. Alternatively, SMAP-1 could directly participate in the assembly of COP-I coatomer.

Additionally, Arf1 has been reported to promote the Golgi association of gamma ear-containing Arf-binding proteins (GGAs) (Doray et al., 2002; D'Souza-Schorey and Chavrier, 2006), which cooperates with clathrin in cargo sorting. In *C. elegans*, APT-9 is the homolog of human GGA1. To determine whether SMAP-1 affects the localization of APT-9/GGA1, we assessed the distribution of APT-9-GFP. In the absence of SMAP-1, although APT-9-GFP accumulated in the cytosol, the localization of APT-9-GFP in mCherry-P4M-labeled Golgi apparatus was significantly reduced (Supplementary Figure S4C-D′). These results suggested that SMAP-1 also functions as a negative regulator of APT-9/GGA1 in *C. elegans* intestinal cells. However, the increase in ARF-1.2 activity does not seem to affect the Golgi association of APT-9/GGA1 directly.

### SMAP-1 Is Localized at the trans-Golgi Network

**FIGURE 2:**
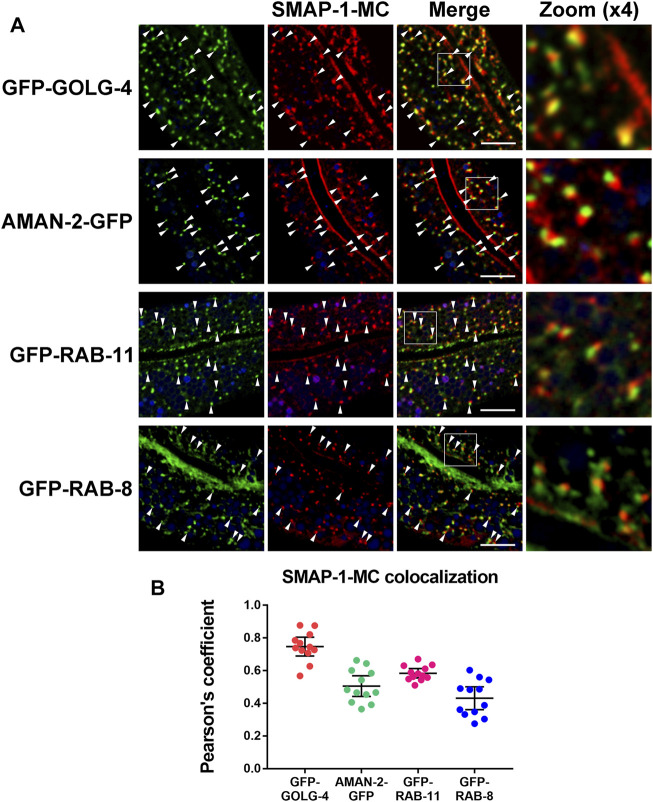
SMAP-1 is localized at the trans-Golgi network. **(A)** SMAP-1 overlapped well with TGN marker GOLG-4/golgin-245. SMAP-1 and *cis-* and *medial-*Golgi marker AMAN-2 were often juxtaposed. SMAP-1 was also juxtaposed to endosome marker RAB-11 and RAB-8. Arrowheads designate structures co-labeled by GFP and mCherry. **(B)** Pearson’s correlation coefficients are calculated, error bars are 95% CIs (n = 12 animals). Scale bars, 10 μm.

To characterize the intracellular position of SMAP-1, we compared mCherry-tagged SMAP-1 with a set of organelle markers. In the wild-type background, SMAP-1 localized to punctate structures in intestinal cells. In agreement with its functional implication, SMAP-1 overlapped with TGN marker GOLG-4/golgin-245 (Figures 2A,B) (Munro, 2011). AMAN-2 (alpha-mannosidase II) labels *cis-* and *medial-*Golgi (Sato et al., 2011). We observed an absence of colocalization between SMAP-1 and AMAN-2 (Figures 2A,B), which were often adjacent. Phosphoinositide PI(4)P is mainly enriched in the Golgi (Dickson et al., 2016). Consistently, SMAP-1 colocalized with PI(4)P marker GFP-P4M in cytosolic punctate structures (Supplementary Figure S5A,B). SMAP-1 was also juxtaposed to exocytosis-associated endosome markers RAB-11 and RAB-8 (Figures 2A,B) (Huber et al., 1993; Ang et al., 2003; Sato et al., 2008; Winter et al., 2012).

### Localization of SMAP-1 in the trans-Golgi Network Requires AP-1

**FIGURE 3:**
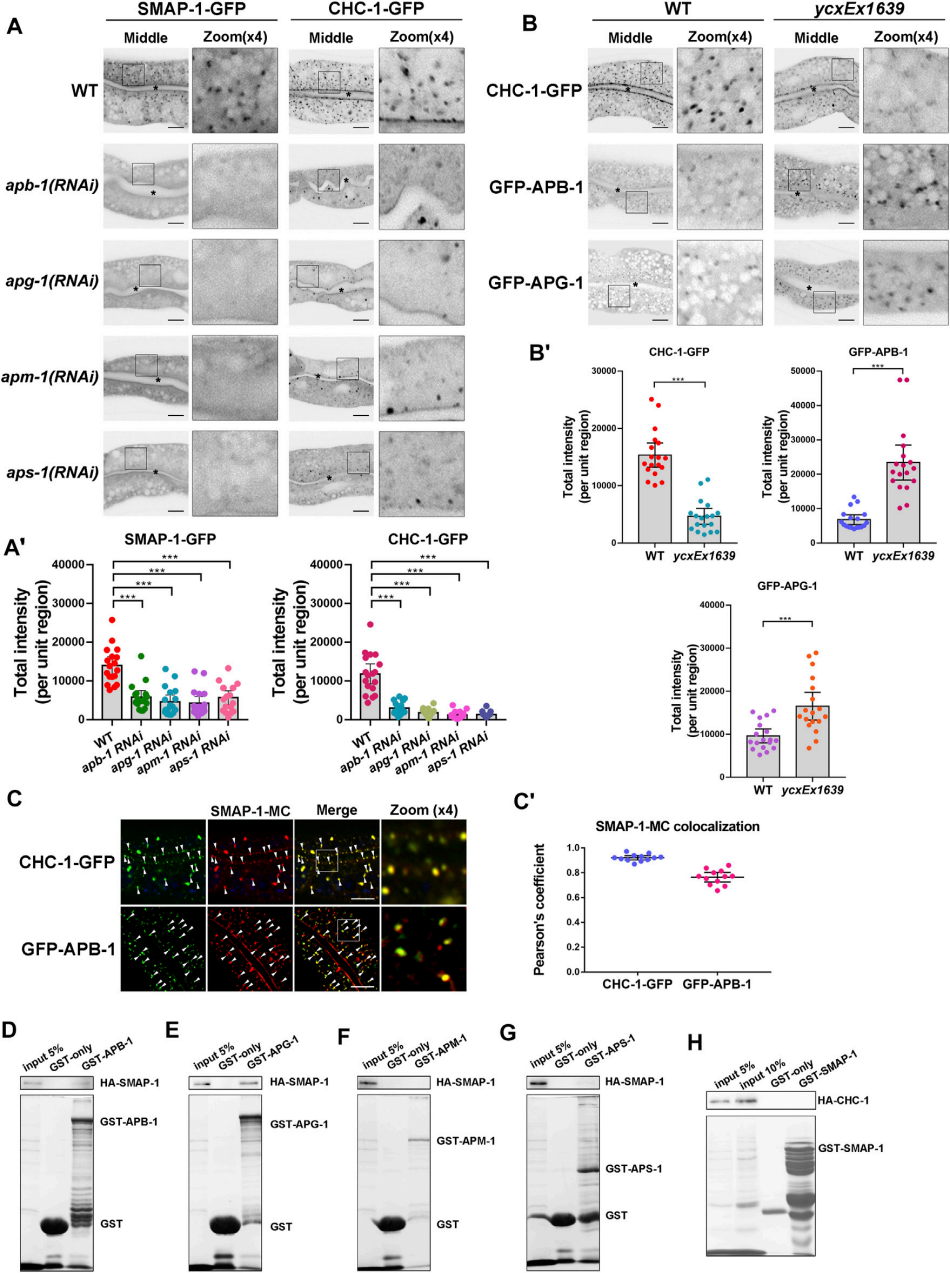
The localization of SMAP-1 in the trans-Golgi network requires AP-1. **(A-A′)** In the middle focal plane, SMAP-1-GFP-labeled structures were reduced upon loss of AP-1 subunits. Also, the punctate structures labeled by CHC-1 (clathrin heavy chain) were decreased in the absence of AP-1 subunits. Error bars are 95% CIs (n = 18 each). Asterisks designate the significant differences in a one-way ANOVA followed by a post-hoc test (Dunn's Multiple Comparison Test) for multiple comparisons (***p < 0.001). **(B-B′)** Loss of SMAP-1 led to the accumulation of GFP-APG-1 and GFP-APB-1 on the punctate structures. In contrast, CHC-1-positive punctate structures were reduced in SMAP-1-deficient cells. Error bars are 95% CIs (n = 18 each). Asterisks designate the significant differences in the Mann-Whitney test (***p < 0.001). **(C-C′)** SMAP-1-mCherry colocalized well with CHC-1 and APB-1. Arrowheads designate structures co-labeled by GFP and mCherry. Pearson’s correlation coefficients are calculated, error bars are 95% CIs (n = 12 animals). **(D–H)** Western blot showing GST pull-down with *in vitro* translated HA-tagged proteins. GST-APG-1 exhibited interactions with HA-SMAP-1. Scale bars, 10 μm.

Previous studies showed that the AP-1 complex mediates clathrin assembly and acts synergistically with clathrin to regulate sorting on the TGN (Robinson and Bonifacino, 2001). The punctate structures labeled by CHC-1 (clathrin heavy chain) were consistently reduced in the absence of AP-1 subunits (Figure 3A-A′). In addition, depleting AP-1 subunits or clathrin caused ERM-1 to accumulate around the basolateral membrane and resulted in the presence of SLCF-1 in the apical membrane of intestinal cells (Supplementary Figure S6A-A′). Remarkably, most SMAP-1-GFP-labeled structures also disappeared upon loss of AP-1 subunits (Figure 3A-A′). It is noteworthy that the *C. elegans* genome encodes an additional AP-1 μ1 subunit UNC-101 (Shim et al., 2000), which has been implicated in the polarized sorting of KVS-4 in DA9 motor neurons (Zhou et al., 2016). Conversely, loss of UNC-101 did not disturb the distributional of CHC-GFP (Supplementary Figure S6C-C′), supporting the distinct, tissue-specific functions of APM-1 and UNC-101 (Shim et al., 2000).

To further determine the genetic relationship between SMAP-1 and AP-1 or clathrin, we examined the distribution of clathrin and the AP-1 complex in *smap-1* mutants. Of note, CHC-1-positive punctate structures decreased in SMAP-1-deficient cells (Figure 3B-B′). Large subunits APG-1 (γ subunit) and APB-1 (β1 subunit) are associated with the membrane and clathrin (Heldwein et al., 2004; Doray et al., 2007). Both GFP-APG-1 and GFP-APB-1 accumulated on the punctate structures in *smap-1* mutants (Figure 3B-B′). Furthermore, we noticed that SMAP-1-mCherry colocalized with CHC-1, APB-1, and APM-1 (μ1 subunit) in intestinal cells (Figure 3C-C′; Supplementary Figure S6B-B′). To determine the interaction between SMAP-1 and AP-1 subunits APM-1 (μ1), APB-1 (β1), APG-1 (γ), and APS-1 (σ1), we performed GST pull-down assays. We found that SMAP-1 was bound to APG-1, while there was no significant interaction between SMAP-1 and APM-1, APB-1, and APS-1 (Figures 3D–G). In contrast, we did not observe the interaction between SMAP-1 and clathrin heavy chain (CHC-1) (Figure 3H). Together, these data suggested that the integrity of the AP-1 complex is required for SMAP-1 localization in TGN and that the clathrin assembly event likely occurs downstream of SMAP-1.

**FIGURE 4:**
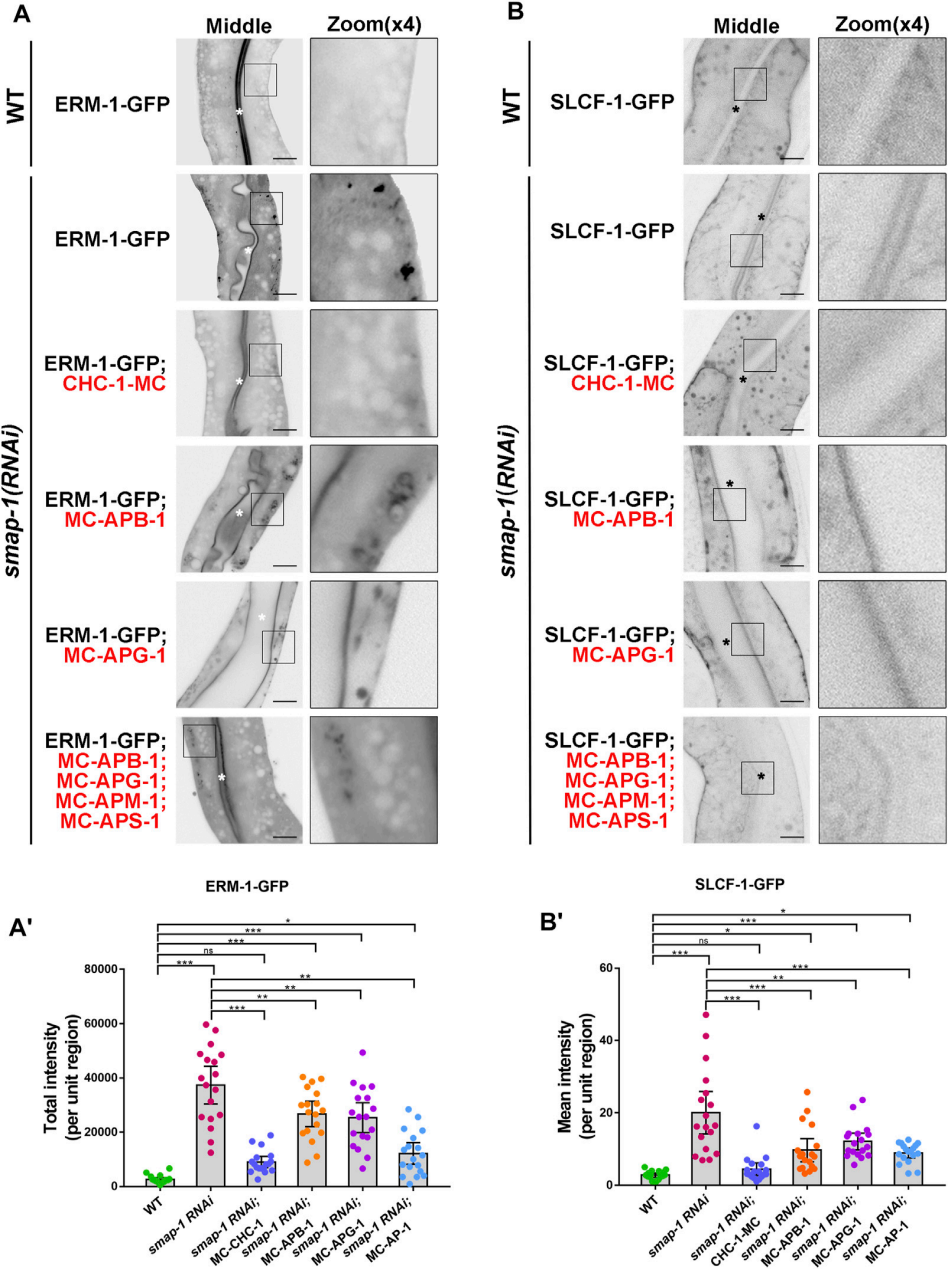
Overexpression of CHC-1 relieved the mislocalization phenotype of ERM-1 and SLCF-1 in SMAP-1 knockdown animals. **(A-A′)** In *smap-1(RNAi)* mutants, overexpressed CHC-1 fully rescued the basolateral mislocalization phenotype of ERM-1. Error bars are 95% CIs (n = 18 each). Asterisks designate the significant differences in a one-way ANOVA followed by a post-hoc test (Dunn's Multiple Comparison Test) for multiple comparisons (***p < 0.001, **p < 0.01, *p < 0.05, ns: no significance). **(B-B′)** In *smap-1(RNAi)* mutants, overexpressed CHC-1 fully rescued the apical mislocalization phenotype of SLCF-1. Error bars are 95% CIs (n = 18 each). Asterisks designate the significant differences in a one-way ANOVA followed by a post-hoc test (Dunn's Multiple Comparison Test) for multiple comparisons (***p < 0.001, **p < 0.01, *p < 0.05, ns: no significance). Scale bars, 10 μm.

Next, we inspected the subcellular distribution of ERM-1-GFP and SLCF-1-GFP in SMAP-1 knockdown animals (Supplementary Figure S2B). As expected, overexpression of CHC-1 rescued the mislocalization phenotype of ERM-1 and SLCF-1 in the case of SMAP-1 deficiency (Figure 4A-B′). Conversely, the simultaneous overexpression of mCherry-tagged APM-1 (μ1), APB-1 (β1), APG-1 (γ), and APS-1 (σ1) failed to fully alleviate distribution defects of cargos in *smap-1(RNAi)* animals (Figure 4A-B′). Hence, our results suggested that SMAP-1 helps couple the AP-1 complex and clathrin in TGN-mediated sorting in the *C. elegans* intestine.

### Loss of SMAP-1 Leads to Reduced Clathrin Coat Assembly in the TGN

**FIGURE 5:**
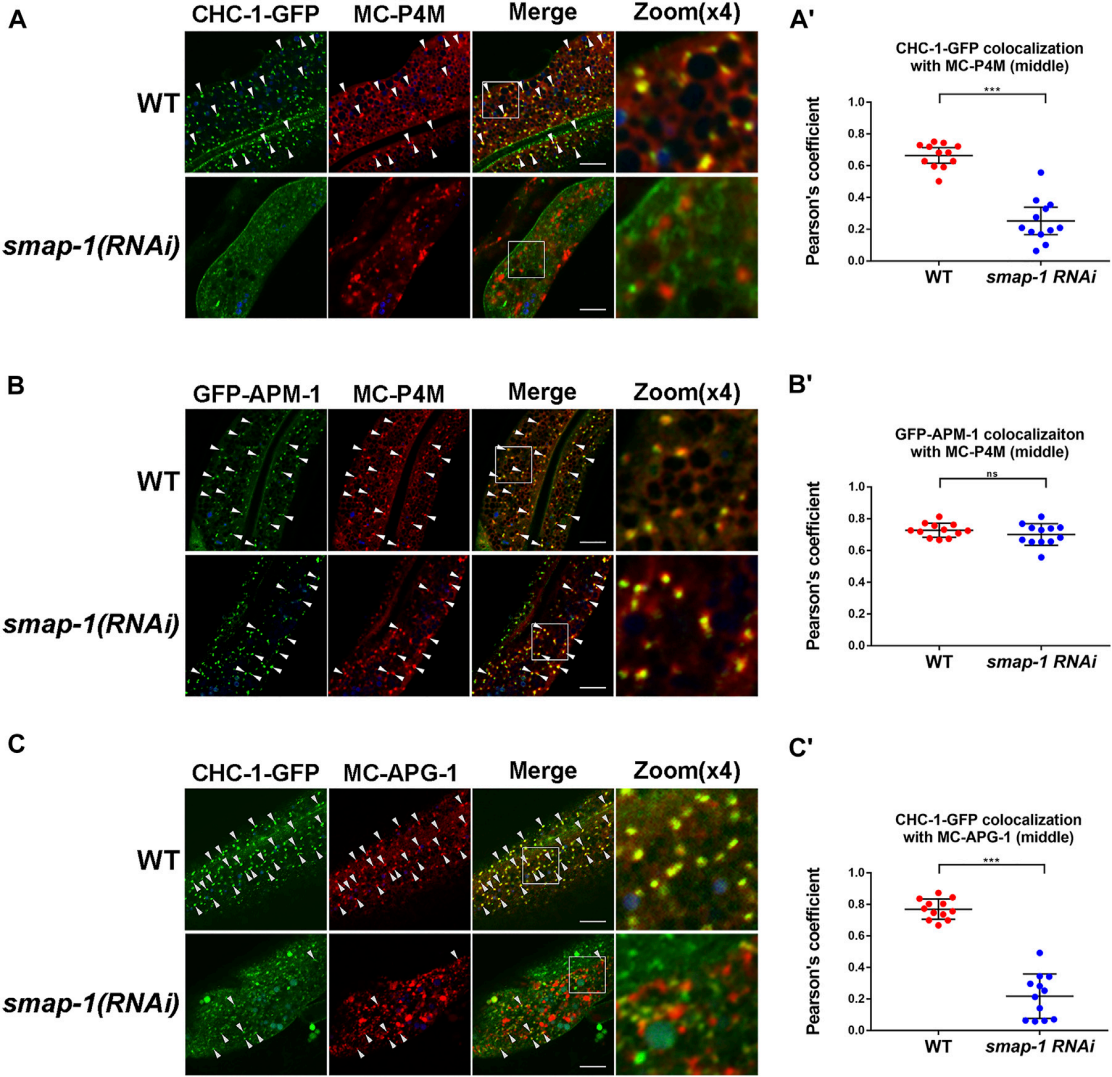
Loss of SMAP-1 leads to a decrease in TGN-located clathrin. **(A-A′)** Loss of SMAP-1 led to a decrease in the colocalization between CHC-1 and P4M. Pearson’s correlation coefficients are calculated, error bars are 95% CIs (n = 12 animals). *p*-value: Mann-Whitney test. ***p < 0.001. **(B-B′)** The colocalization between APM-1 and P4M was not affected by the depletion of SMAP-1. Pearson’s correlation coefficients are calculated, error bars are 95% CIs (n = 12 animals). *p*-value: Mann-Whitney test. ns: no significance. **(C-C′)** In the absence of SMAP-1, CHC-1 failed to overlap with APG-1 in punctate structures. Pearson’s correlation coefficients are calculated, error bars are 95% CIs (n = 12 animals). *p*-value: Mann-Whitney test. ***p < 0.001. Scale bars, 10 μm.

Thus far, our analysis revealed that clathrin assembly is likely to occur downstream of SMAP-1. To further clarify the effect of SMAP-1 on clathrin localization, we compared clathrin with the PI(4)P marker GFP-P4M in the absence of SMAP-1. Remarkably, loss of SMAP-1 reduced the overlap between residual CHC-1-GFP and mCherry-P4M (Figure 5A-A′), validating that SMAP-1/SMAP2 regulates the occurrence of clathrin in the Golgi apparatus. However, the Golgi localization of APM-1 was not affected upon loss of SMAP-1 (Figure 5B-B′). We subsequently examined the level of colocalization between CHC-1-GFP and mCherry-APG-1. In the absence of SMAP-1, we found that the remaining CHC-1-labeled structure no longer colocalized with APG-1 (Figure 5C-C′). In addition to biosynthetic sorting, the clathrin coat is known to mediate the formation of endocytic clathrin-coated vesicles (Chen and Schmid, 2020; Moulay et al., 2020). Also, clathrin has been reported to function as a component of the retrograde transport machinery on the surface of the endosome (Saint-Pol et al., 2004; Shi et al., 2009). Therefore, the punctate structures distinct from the P4M- or APG-1-positive puncta are likely clathrin-coated vesicles and sorting endosomes (Figure 5A-A′, C-C′). Taken together, our results indicated that SMAP-1 acts as an indispensable regulator that directs TGN clathrin coat assembly downstream of the AP-1 complex.

## Discussion

Here, we identified SMAP-1/SMAPs as a polarized secretion regulator in the *C. elegans* intestine. SMAP-1 colocalizes with AP-1 and clathrin in the TGN. The integrity of the AP-1 complex is required for SMAP-1 positioning, and SMAP-1 acts to sustain clathrin assembly to ensure AP-1/clathrin-dependent cargos sorting (Supplementary Figure S7).

Studies in mammals indicated that SMAP1 functions as an Arf6GAP to regulate clathrin-dependent endocytosis via binding directly to clathrin (Tanabe et al., 2005). Additionally, SMAP2 was implicated in endosome-to-Golgi retrograde transport (Natsume et al., 2006). A recent study showed that SMAP2 facilitates clathrin assembly protein (CALM) mediated formation of clathrin-coated carriers on the TGN, promoting acrosome formation (Funaki et al., 2013). Together, these results suggested that SMAPs are clathrin assembly regulators in the TGN, and this efficacy could be due to its ArfGAP activity. In the current study, we found that SMAP-1 regulates polarized sorting, and this function seems independent of ARF-1.2, supporting the role of SMAPs as secretion regulators. Furthermore, our study highlighted the diversity of SMAPs functionality and corroborated the significance of AP-1/clathrin coat assembly in polarized sorting.

Previous studies have shown that SMAPs interact with clathrin and CALM, modulating clathrin-coated vesicle formation on the TGN (Tanabe et al., 2005; Natsume et al., 2006; Funaki et al., 2013). However, the mechanism controlling the localization of SMAPs is still not well understood. Here, by using a well-established *in vivo* membrane trafficking investigation model (Chen et al., 2018; Chen et al., 2019; Gao et al., 2020; Zhang et al., 2020; Yan et al., 2021), we showed that the integrity of AP-1 adaptor is necessary for the TGN positioning of SMAP-1. Although we did not specifically identify which AP-1 subunit governs the TGN localization of SMAP-1, our results suggested that in addition to CALM, SMAP-1 underlies an additional clathrin assembly mechanism, enriching the understanding of AP-1/clathrin coat assembly. It is reasonable to speculate that a similar mechanism might be involved in the budding of clathrin-coated vesicles during endocytosis. Further analyses are required to dissect the details of this biological process.

## Materials and Methods

### *C. elegans* Strains

Genetic crosses of *C. elegans* were performed by standard methods (Brenner, 1974). A list of strains was provided in Supplemental Materials. RNAi-mediated gene expression interference was implemented by the feeding protocol (Timmons and Fire, 1998). RNAi constructs were from the Ahringer library (Kamath and Ahringer, 2003). For *chc-1*, *apb-1*, *apg-1*, *apm-1*, and *aps-1* RNAi experiments, L2-L3 stage larvae were cultured for 48–60h and scored as adults.

### Antibodies

Mouse anti-α-Tubulin monoclonal antibody (T6199, Sigma, St. Louis, MO), and mouse anti-Flag monoclonal antibody (F1804; Sigma, St. Louis, MO) were used in this study.

### CRISPR-Cas9 Mutant Strains

The CRISPR/Cas9 vectors were assembled by swapping the eft-3 promoter in pDD162 (Addgene, #47549) with the heat-shock promoter P*hsp*-16.2 (Shen et al., 2014; Li et al., 2015). CRISPR design tool (https://chopchop.cbu.uib.no/) was used to identify the knockout targets. Three *smap-1* target sequences were selected, including AGG​TAA​AGT​GGA​TCC​GAA​GAa​gg, AAG​ACT​GCA​AGG​CTT​CCT​GTt​gg, AAA​AGT​GCG​CTC​AGT​GAA​TCt​gg. The CRISPR/Cas9 plasmids were validated by sequencing. CRISPR/Cas9 conditional knockout strains were created by microinjection of plasmids at 50 ng/μl and Podr-1:rfp (50 ng/μl) into wild-type hermaphrodites germline (Zhou et al., 2016). Heat-shock was executed at 0 h, 8 h, 16 h, 24 h, and 32 h after egg-hatching. The apical membrane cargo SLCF-1-GFP showed significant defects after heat shock at 0 h.

### Plasmids and Transgenic Strains

For the SMAP-1 (R60A) rescue assay, a guide RNA (sgRNA) resistant plasmid was prepared by introducing silent mutations into each target sequence (5′-AGG​CAA​GGT​TGA​CCC​AAA​AAa​ag-3′, 5′-GAG​ACT​ACA​GGG​ATT​TCT​ATt​ag-3′, 5′-GAA​GGT​ACG​TTC​TGT​TAA​CCt​ag-3′). We also introduced a single missense mutation in the SMAP-1 GAP domain. To construct transgenes expressed explicitly in *C. elegans* intestine, the intestine-specific promoter *vha-6* driven vectors modified with a Gateway cassette were deployed. The cDNA sequences of *smap-1(w09d10.1)*, *erm-1*, *slcf-1*, *nhx-2*, *chc-1*, *apb-1*, *apg-1*, *apm-1*, *aps-1*, *golg-4*, *P4M*, and *arf-1.2* lacking a stop codon or a start codon were cloned into intestinal vectors by LR reaction (Chen et al., 2006). Transgenic *strains* were generated by standard microinjection; plasmids were co-injected with selection markers P*odr-1:gfp* or P*odr-1:rfp* into wild-type or *smap-1(ycxEx1639)* hermaphrodites germ lines.

### Worm Lysate Preparation and Western Blot

Around 100 wild-type or *smap-1(RNAi)* young adults (24 h after L4 stage) were picked into 20 μl lysis buffer [100 mM Tris pH 6.8, 8% SDS, 20 mM β-mercaptoethanol], then mixed with 20 μl 2xSDS-PAGE loading buffer and boiled at 100 °C for 10min. Lysates were resolved on SDS-PAGE [12% (wt/vol) polyacrylamide], blotted to nitrocellulose. After 5% milk blocking and washing, the membrane was blotted with anti-Flag and anti-Tubulin antibodies.

### Microscopy and Image Analysis

Live animals were mounted on 2% agarose pads (100 mM levamisole). Fluorescence images were obtained with a Nikon C2 laser scanning confocal microscope (Nikon, Tokyo, Japan) equipped with a 100×N.A. 1.2 oil-immersion objective. Images were collected with NIS-Elements AR 4.40.00 software. Z-series of optical sections were acquired using 0.8–1 μm step size. Fluorescence data were evaluated with Metamorph software version 7.10.3.279 (Universal Imaging, West Chester, PA). The “Integrated Morphometry Analysis” component was utilized to assess the fluorescence intensity (total intensity), puncta number (structure count), and fluorescence area (total area) within unit regions. For each genotype, a total of 6 animals were analyzed in three unit regions of each intestine defined by a 100 × 100 (pixl^2^) box located randomly (n = 18 each). In this case, “total area” is a comprehensive parameter indicating the number and size of the fluorescent structures. Colocalization images were analyzed by Fiji (Image J) software (Schindelin et al., 2012). Pearson’s correlation coefficients were calculated with 6 animals for all genotypes.

### Statistical Analysis

Prism software version 8.02 (GraphPad Software, La Jolla, CA) was deployed to perform statistical analyses.

## Data Availability

The original contributions presented in the study are included in the article/Supplementary Material, further inquiries can be directed to the corresponding author.

## Supplementary Material

Supplementary Figure S1 Differential interference contrast (DIC) and fluorescence images showing that SMAP-1 is expressed in multiple tissues of *C. elegans*. **(A)** A GFP transgene driven by the *smap-1* promoter in an adult hermaphrodite. **(B)** In the tail, arrows indicate neuron and intestine. **(C)** In the posterior region, arrows indicate intestine and ventral nerve cord (VNC). **(D)** In the head, arrowhead indicates pharynx. **(E)** In the mid-body, arrows indicate VNC and dorsal nerve cord (DNC). Scale bars, 10 μm.

Supplementary Figure S2
**(A-A′)** Confocal images showing SLCF-1-GFP distribution in the intestinal cells. Heat-shock was executed at 0, 8, 16, 24, and 32 h after hatching. The SLCF-1-GFP localization was examined in young adult animals. Error bars are 95% CIs (*n* = 18 each, 9 animals of each genotype were sampled in whole-cell regions of two intestinal cells). Asterisk indicates the significant difference in a one-way ANOVA followed by a post-hoc test (Dunn's Multiple Comparison Test) for multiple comparisons (****p* < 0.001, ns: no significance). **(B)** Western blot showing Flag-SMAP-1 levels in wild type and *smap-1(RNAi)* backgrounds. Scale bars, 10 μm.

Supplementary Figure S3
**(A-A′)** Confocal images showing the subcellular localization of ARF-1.2-GFP. Black asterisks in the panels indicate intestinal lumen. Error bars are 95% CIs (*n* = 18 each, 9 animals of each genotype were sampled in whole-cell regions of two intestinal cells). Asterisks indicate the significant differences in the Mann-Whitney test (****p* < 0.001, **p* < 0.05). **(B-B′)** The membrane-to-cytosol ratio (P/S) of ARF-1.2-GFP increased in *smap-1(RNAi)* animals. Membrane structures were separated from the cytosol of worm lysates by ultracentrifugation. ARF-1.2-GFP in the supernatants and pellets were analyzed by western blotting using an anti-GFP antibody. The loading control was blotted by the anti-Tubulin antibody. The SEMs from three independent experiments are shown, asterisk indicates the significant differences in the one-tailed Student’s t-test (****p* < 0.001). **(C-C′)** Confocal images of the intestinal cells expressing GFP-tagged ERM-1 and SLCF-1. Asterisks in the panels indicate intestinal lumen. Error bars are 95% CIs (*n* = 18 each, 9 animals of each genotype were sampled in whole-cell regions of two intestinal cells). Asterisks indicate the significant differences in the Mann-Whitney test (****p* < 0.001, ns: no significance). **(D-D′)** Confocal images of the intestinal cells expressing GFP-tagged ERM-1 and SLCF-1. In *smap-1(ycxEx1639)* mutants, overexpression of SMAP-1(R60A)-mCherry that has lost GAP activity rescued the distribution defects of ERM-1-GFP and SLCF-1-GFP. Asterisks in the panels indicate intestinal lumen. Error bars are 95% CIs (*n* = 18 each, 9 animals of each genotype were sampled in whole-cell regions of two intestinal cells). Asterisks indicate the significant differences in the Mann-Whitney test (****p* < 0.001, ns: no significance). Scale bars, 10 μm.

Supplementary Figure S4
**(A-A′)** Confocal images showing colocalization between COPG-1 and Golgi marker P4M in the intestinal cells. Arrowheads indicate structures labeled by both GFP and mCherry. Pearson’s correlation coefficients for GFP and mCherry signals are calculated, error bars are 95% CIs (*n* = 12 animals). P-value: Mann-Whitney test. ****p* < 0.001. **(B-B′)** Confocal images showing colocalization between COPB-1 and Golgi marker P4M in the intestinal cells. Arrowheads indicate structures labeled by both GFP and mCherry. Pearson’s correlation coefficients for GFP and mCherry signals are calculated, error bars are 95% CIs (*n* = 12 animals). P-value: Mann-Whitney test. ****p* < 0.001. **(C-C′)** Confocal images showing colocalization between APT-9 and Golgi marker P4M in the intestinal cells. Arrowheads indicate structures labeled by both GFP and mCherry. Pearson’s correlation coefficients for GFP and mCherry signals are calculated, error bars are 95% CIs (*n* = 12 animals). P-value: Mann-Whitney test. ****p* < 0.001. **(D-D′)** Confocal images showing APT-9-GFP in the intestinal cells. Error bars are 95% CIs (*n* = 18 each, 9 animals of each genotype were sampled in whole-cell regions of two intestinal cells). Asterisks indicate the significant differences in the Mann-Whitney test (****p* < 0.001). Scale bars, 10 μm.

Supplementary Figure S5
**(A)** Confocal image showing colocalization between PI(4)P marker GFP-P4M and SMAP-1-mCherry in the intestinal cells. Arrowheads indicate structures labeled by both GFP and mCherry. **(B)** Pearson’s correlation coefficients for GFP and mCherry signals are calculated, error bars are 95% CIs (*n* = 12 animals). Scale bar represents 10 μm.

Supplementary Figure S6
**(A-A′)** Confocal images of the intestinal cells expressing GFP-tagged ERM-1 and SLCF-1. Asterisks in the panels indicate intestinal lumen. Error bars are 95% CIs (*n* = 18 each, 9 animals of each genotype were sampled in whole-cell regions of two intestinal cells). Asterisks indicate the significant differences in a one-way ANOVA followed by a post-hoc test (Dunn's Multiple Comparison Test) for multiple comparisons (****p* < 0.001). **(B-B′)** Confocal image showing colocalization between GFP-APM-1 (μ1 subunit) and SMAP-1-mCherry in the intestinal cells. Arrowheads indicate structures labeled by both GFP and mCherry. Pearson’s correlation coefficients for GFP and mCherry signals are calculated, error bars are 95% CIs (*n* = 12 animals). **(C-C′)** Confocal images showing CHC-1-GFP in the intestinal cells. Error bars are 95% CIs (*n* = 18 each, 9 animals of each genotype were sampled in whole-cell regions of two intestinal cells). Asterisks indicate the significant differences in the Mann-Whitney test (ns: no significance). Scale bars, 10 μm.

Supplementary Figure S7 In the *C. elegans* intestine, ARF-1.2 regulates clathrin coat assembly via downstream AP-1 and SMAP-1, promoting TGN sorting. In addition, SMAP-1 acts as a GAP to regulate ARF-1.2 activity in a negative feedback manner.
