# Supplementary figures and images for: AP-1 Recruits SMAP-1/SMAPs to the trans-Golgi Network to Promote Sorting in Polarized Epithelia
Source: Front Cell Dev Biol. 2021 Nov 25;9:774401. doi: 10.3389/fcell.2021.774401 (PMC8655793; doi:10.3389/fcell.2021.774401)

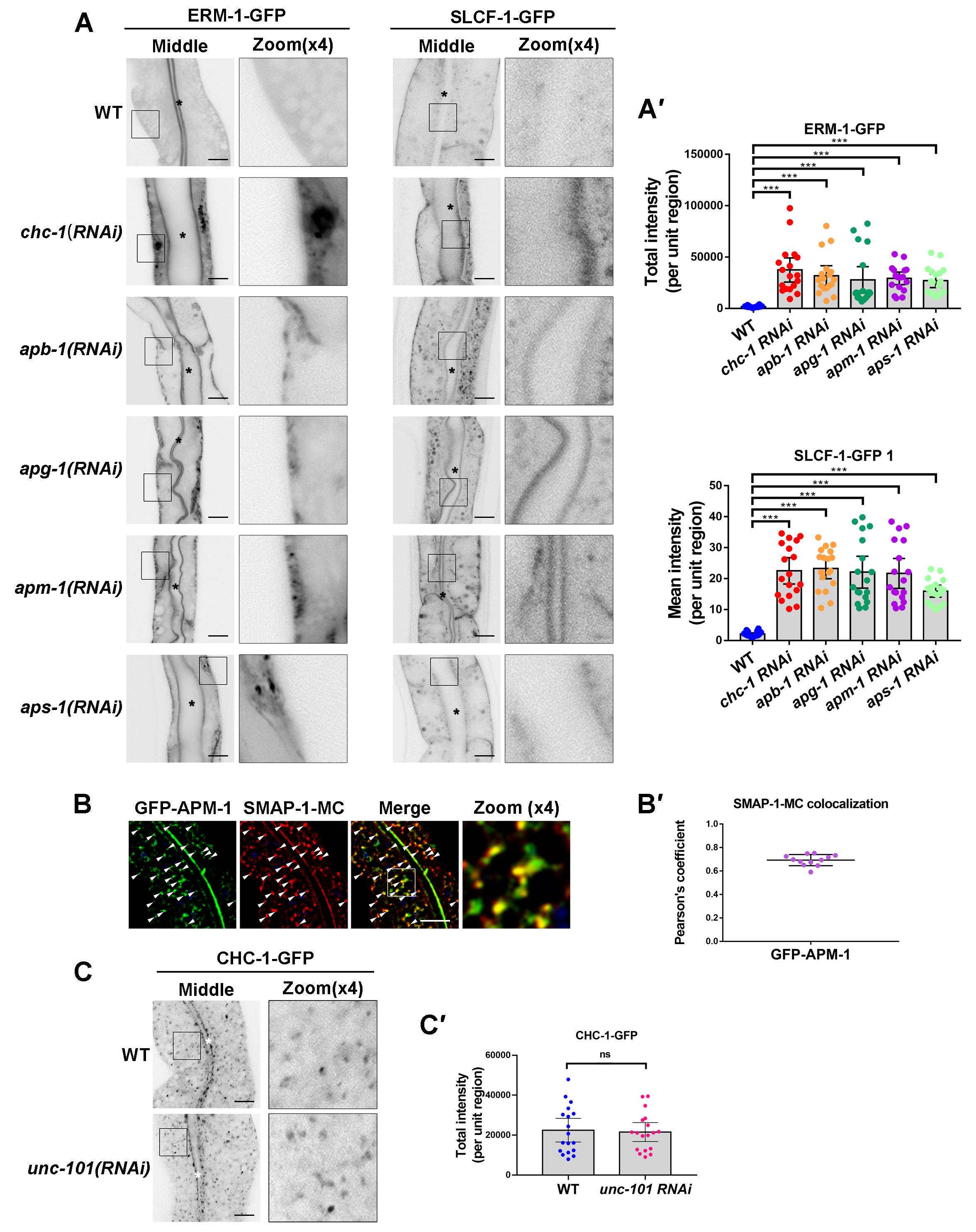

Supplement: Supplementary file 1 [file Image6.TIF]

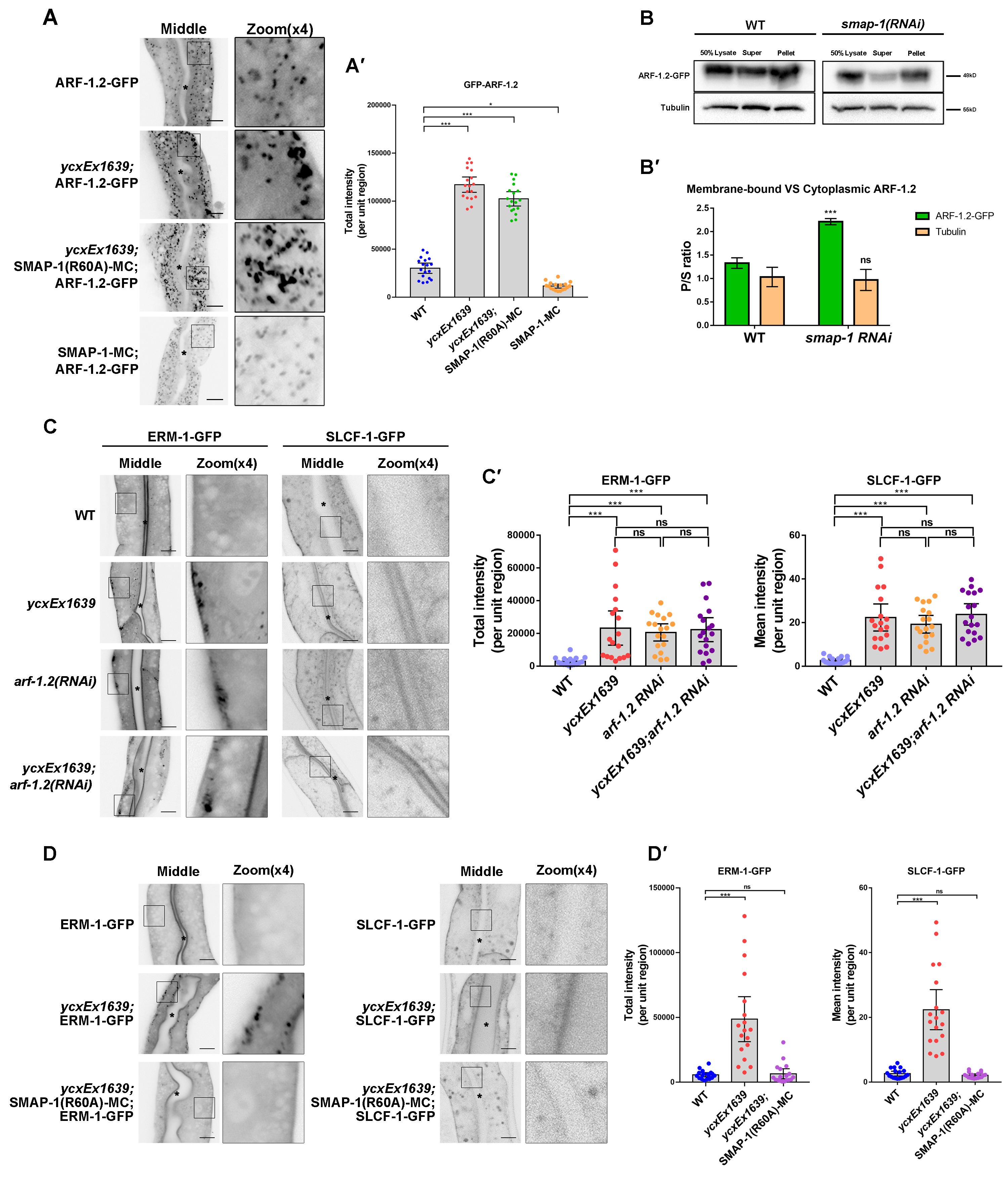

Supplement: Supplementary file 3 [file Image3.TIF]

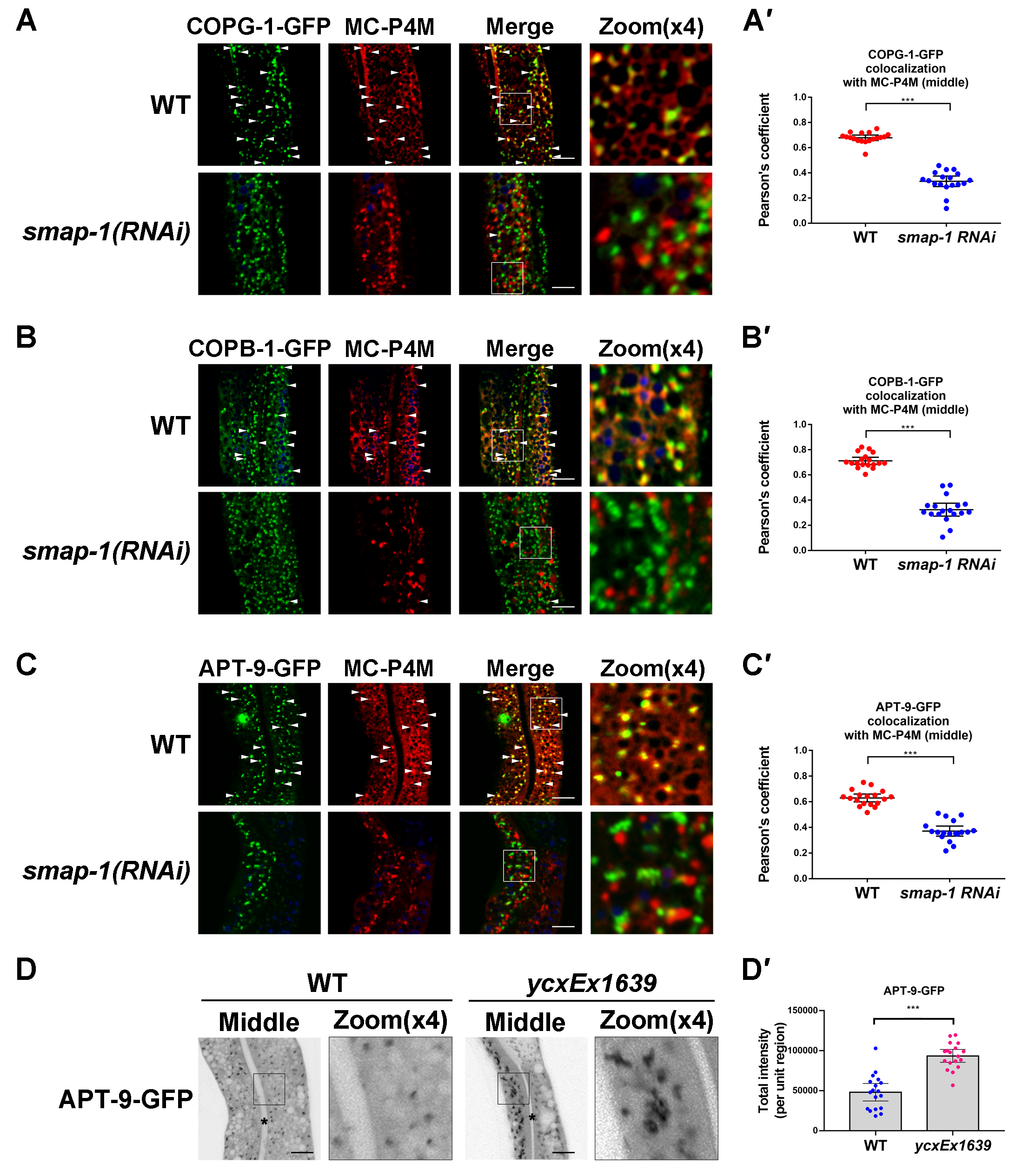

Supplement: Supplementary file 4 [file Image4.TIF]

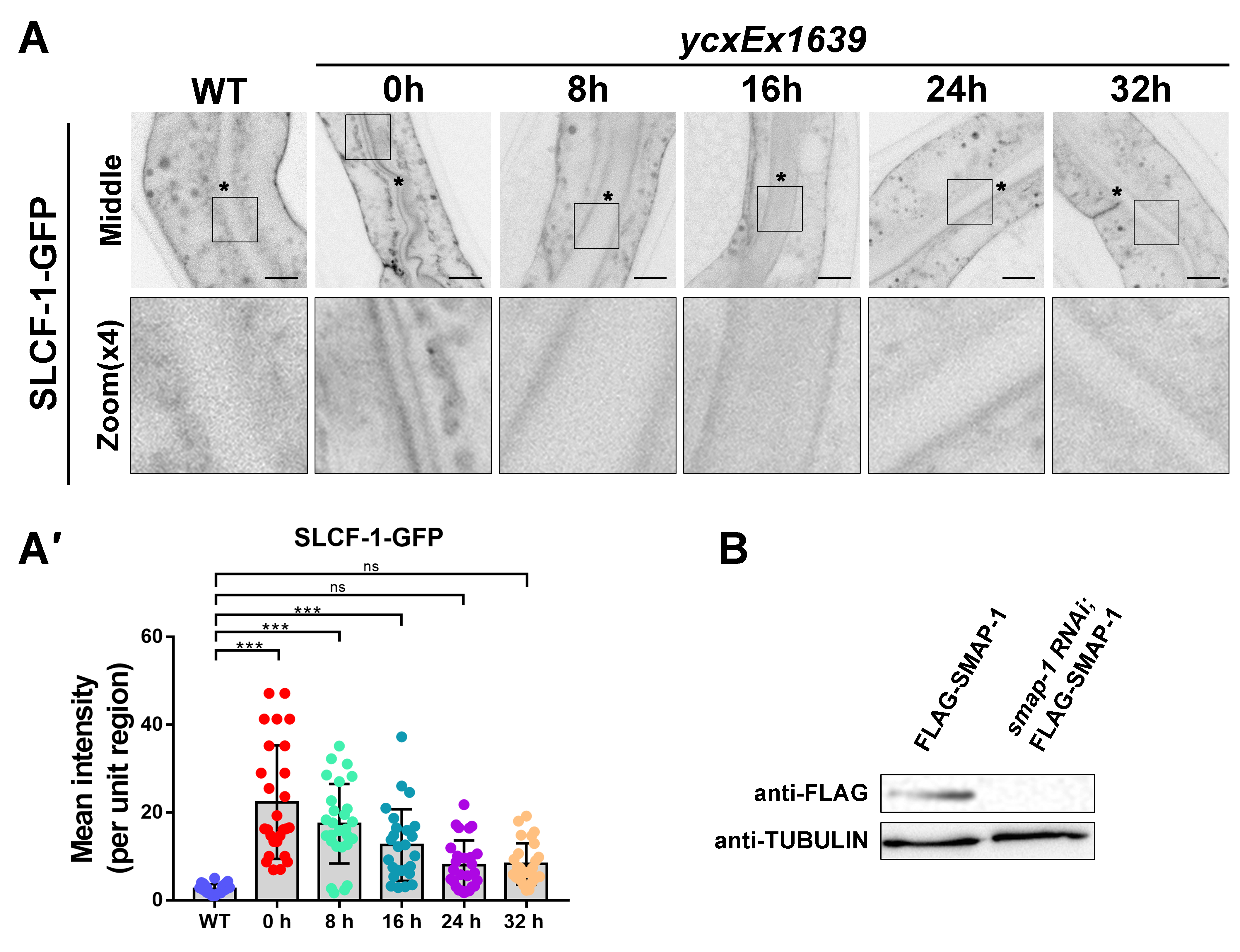

Supplement: Supplementary file 5 [file Image2.TIF]

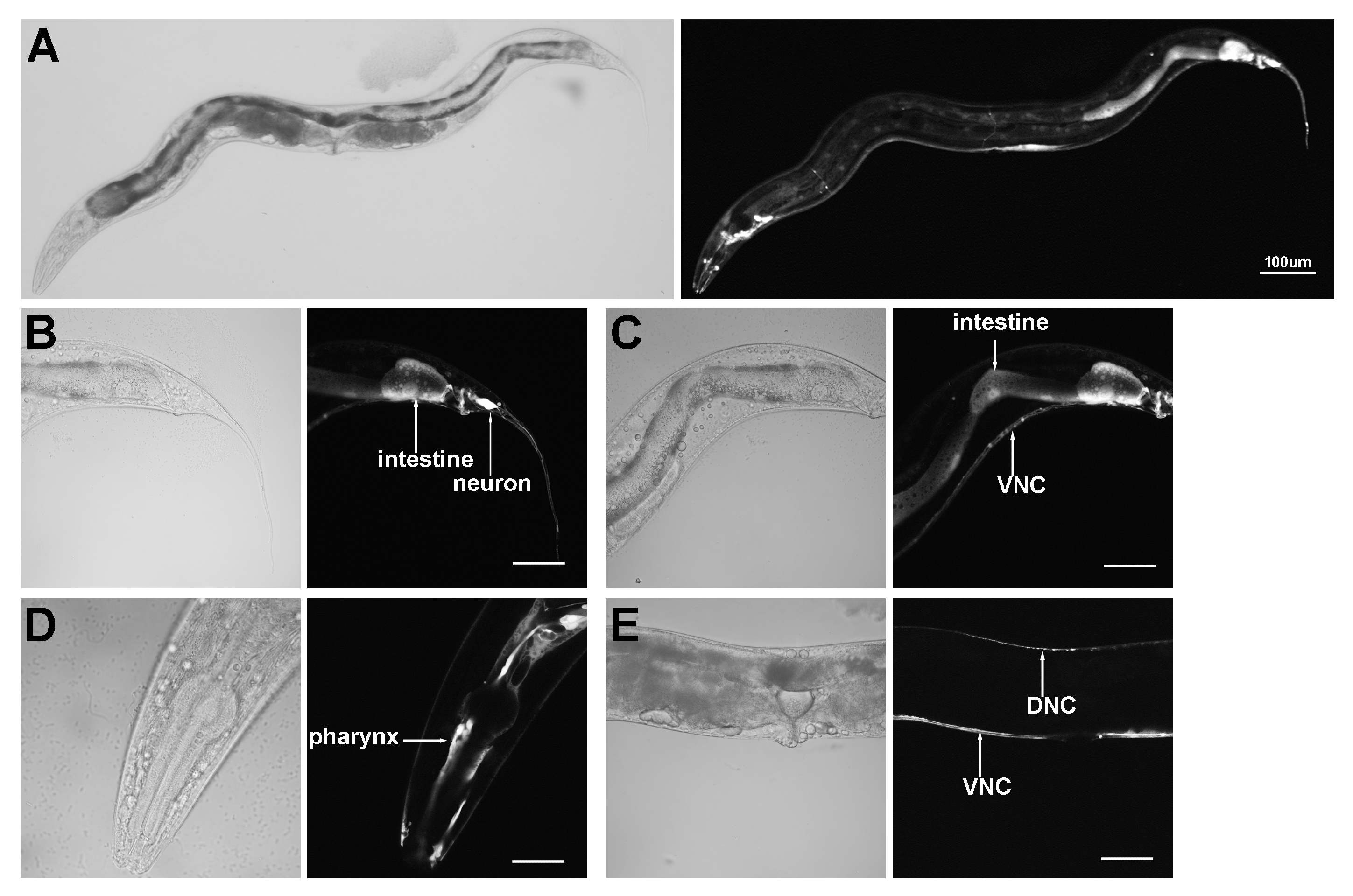

Supplement: Supplementary file 6 [file Image1.TIF]

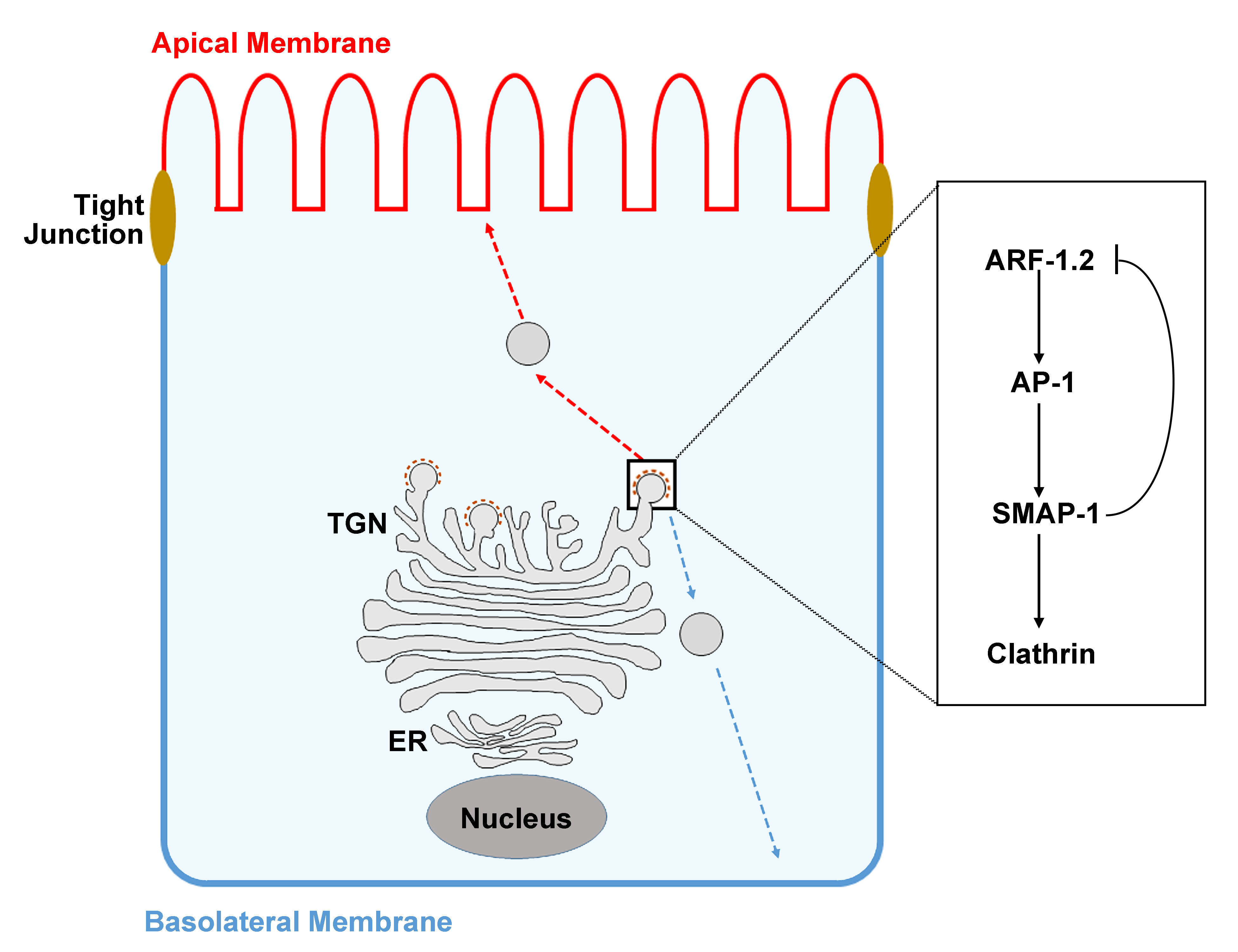

Supplement: Supplementary file 7 [file Image7.TIF]

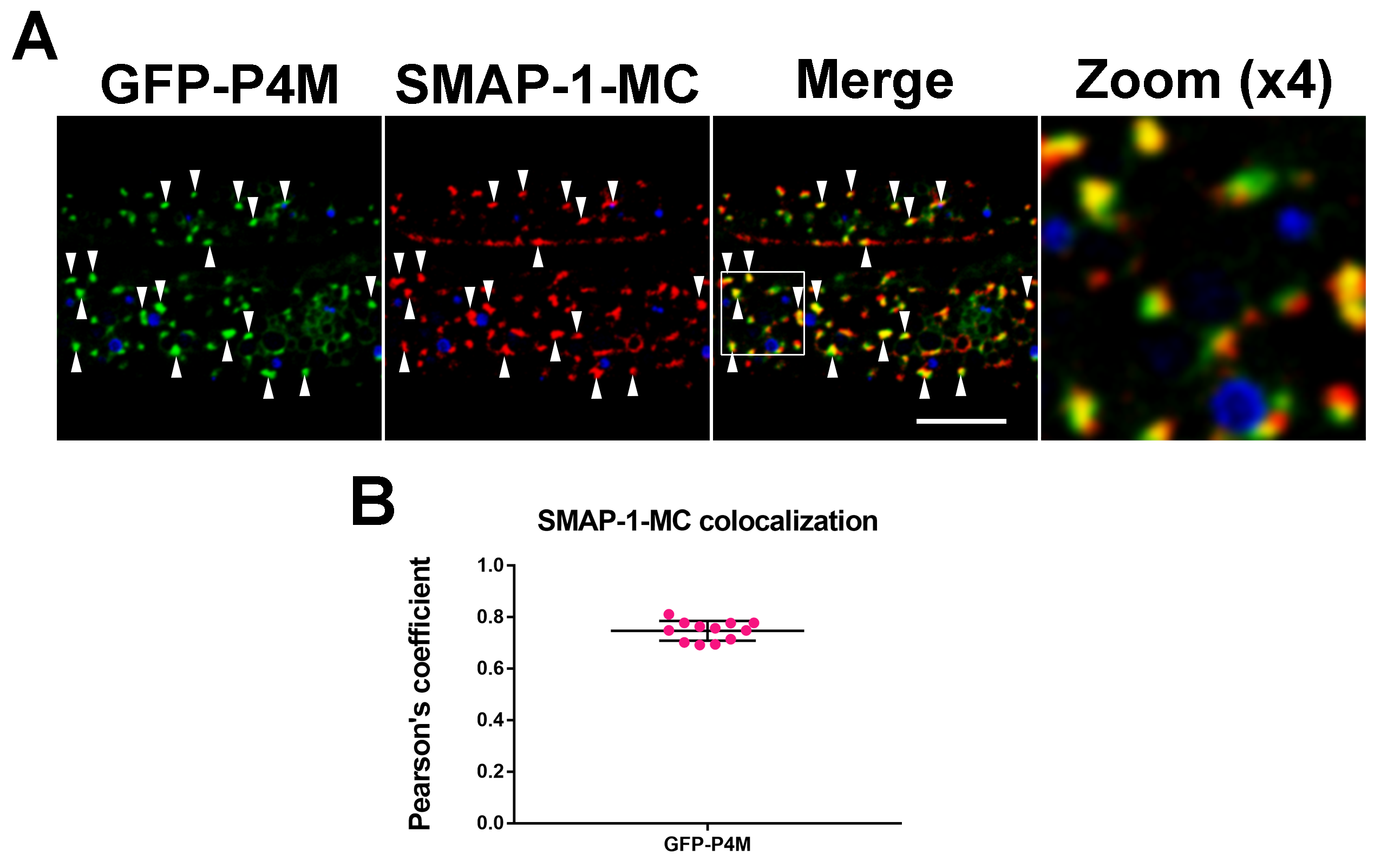

Supplement: Supplementary file 8 [file Image5.TIF]
