# Supplementary material for: AP-1 Recruits SMAP-1/SMAPs to the trans-Golgi Network to Promote Sorting in Polarized Epithelia
Source: Front Cell Dev Biol. 2021 Nov 25;9:774401. doi: 10.3389/fcell.2021.774401 (PMC8655793; doi:10.3389/fcell.2021.774401)
Supplement: Supplementary file 2 [file Table1.DOCX]

| **Table S1. *C. elegans* strains** | | | |
| --- | --- | --- | --- |
| **Strain ID** | **Genotype** | **Method** | **Resource** |
| N2 | Wild type | - | CGC |
| HUS8910 | *Pvha-6::ERM-1::GFP* | Microinjection | This study |
| HUS8911 | *Pvha-6::SLCF-1::GFP* | Microinjection | This study |
| HUS8912 | *HUS8910[ Pvha-6::ERM-1::GFP];HUS5033 [Phsp16.2::Cas9::tbb-2 3’+PU6-SMAP-1::sgRNA I&II&III, Podr-1::gfp]* | Genetic cross | This study |
| HUS8913 | *HUS8911[Pvha-6::SLCF-1::GFP];HUS5033 [Phsp16.2::Cas9::tbb-2 3’+PU6-SMAP-1::sgRNA I&II&III, Podr-1::gfp]* | Genetic cross | This study |
| HUS5033 | *ycxEx1639[Phsp16.2::Cas9::tbb-2 3’+PU6-SMAP-1::sgRNA I&II&III, Podr-1::gfp]* | Genetic cross | This study |
| HUS6021 | *HUS5603 [Pvha-6::GFP::GOLG-4]; HUS5252[Pvha-6-SMAP-1::mCherry]* | Genetic cross | This study |
| HUS4290 | *RT1242[Pvha-6::AMAN-2::GFP]; HUS5252[Pvha-6-SMAP-1::mCherry]* | Genetic cross | This study |
| HUS5481 | *RT311[Pvha-6::GFP::RAB-11]; HUS5252[Pvha-6-SMAP-1::mCherry]* | Genetic cross | This study |
| HUS8914 | *RT310[Pvha-6::GFP::RAB-8]; HUS5252[Pvha-6-SMAP-1::mCherry]* | Genetic cross | This study |
| HUS2558 | *Pvha-6::SMAP-1::GFP* | Microinjection | This study |
| HUS2310 | *Pvha-6::CHC-1::GFP* | Microinjection | This study |
| HUS6436 | *Pvha-6::GFP::APB-1* | Microinjection | This study |
| HUS6453 | *Pvha-6::GFP::APG-1* | Microinjection | This study |
| HUS6410 | *HUS2310[Pvha-6::CHC-1::GFP]; HUS5252[Pvha-6-SMAP-1::mCherry]* | Genetic cross | This study |
| HUS6679 | *HUS6436[Pvha-6::GFP::APB-1]; HUS5252[Pvha-6-SMAP-1::mCherry]* | Genetic cross | This study |
| HUS8915 | *HUS8910[Pvha-6::ERM-1::GFP]; HUS6654[Pvha-6::CHC-1::mCherry]* | Genetic cross | This study |
| HUS8916 | *HUS8910[Pvha-6::ERM-1::GFP]; HUS6437[Pvha-6::mCherry::APB-1]* | Genetic cross | This study |
| HUS8917 | *HUS8910[Pvha-6::ERM-1::GFP]; HUS6454[Pvha-6::mCherry::APG-1]* | Genetic cross | This study |
| HUS8918 | *HUS8911[Pvha-6::SLCF-1::GFP]; HUS6654[Pvha-6::CHC-1::mCherry]* | Genetic cross | This study |
| HUS8919 | *HUS8911[Pvha-6::SLCF-1::GFP]; HUS6437[Pvha-6::mCherry::APB-1]* | Genetic cross | This study |
| HUS8920 | *HUS8911[Pvha-6::SLCF-1::GFP]; HUS6454[Pvha-6::mCherry::APG-1]* | Genetic cross | This study |
| HUS8922 | *HUS2310[Pvha-6::CHC-1::GFP]; HUS8921[Pvha-6::mCherry::P4M]* | Genetic cross | This study |
| HUS8923 | *HUS6275[Pvha-6::GFP::APM-1]; HUS8921[Pvha-6::mCherry::P4M]* | Genetic cross | This study |
| HUS6479 | *HUS2310[Pvha-6::CHC-1::GFP]; HUS6454[Pvha-6::mCherry::APG-1]* | Genetic cross | This study |
| HUS8924 | *Psmap-1::GFP* | Microinjection | This study |
| HUS8925 | *Pvha-6::3xFLAG::SMAP-1* | Microinjection | This study |
| HUS5312 | *HUS4381[Pvha-6::ARF-1.2::GFP]; HUS5033 [Phsp16.2::Cas9::tbb-2 3’+PU6-SMAP-1::sgRNA I&II&III, Podr-1::gfp]* | Genetic cross | This study |
| HUS8926 | *HUS8910[ Pvha-6::ERM-1::GFP]; HUS5033 [Phsp16.2::Cas9::tbb-2 3’+PU6-SMAP-1::sgRNA I&II&III, Podr-1::gfp]; HUS8929 [Pvha-6::SMAP-1(R60A)::mCherry]* | Genetic cross | This study |
| HUS8927 | *HUS8911[Pvha-6::SLCF-1::GFP]; HUS5033 [Phsp16.2::Cas9::tbb-2 3’+PU6-SMAP-1::sgRNA I&II&III, Podr-1::gfp]; HUS8929 [Pvha-6::SMAP-1(R60A)::mCherry]* | Genetic cross | This study |
| HUS8928 | *HUS5107[Pvha-6::GFP::P4M]; HUS5252[Pvha-6::SMAP-1::mCherry]* | Genetic cross | This study |
| HUS6417 | *HUS5298[Pvha-6::GFP::APM-1] ; HUS5252[Pvha-6-SMAP-1::mCherry]* | Genetic cross | This study |
| HUS8906 | *HUS2310[Pvha-6::CHC-1::GFP]; HUS5033 [Phsp16.2::Cas9::tbb-2 3’+PU6-SMAP-1::sgRNA I&II&III, Podr-1::gfp]* | Genetic cross | This study |
| HUS8943 | *HUS6436[Pvha-6::GFP::APB-1] ; HUS5033 [Phsp16.2::Cas9::tbb-2 3’+PU6-SMAP-1::sgRNA I&II&III, Podr-1::gfp]* | Genetic cross | This study |
| 8986 | *HUS6453[Pvha-6::GFP::APG-1] ; HUS5033 [Phsp16.2::Cas9::tbb-2 3’+PU6-SMAP-1::sgRNA I&II&III, Podr-1::gfp]* | Genetic cross | This study |
| HUS6769 | *Pvha-6::NHX-2::GFP* | Microinjection | This study |
| HUS8983 | *HUS6769[Pvha-6::NHX-2::GFP]; HUS5033 [Phsp16.2::Cas9::tbb-2 3’+PU6-SMAP-1::sgRNA I&II&III, Podr-1::gfp]* | Genetic cross | This study |
| HUS1435 | *Pvha-6::LET-413::GFP* | Microinjection | Liu et al., 2017 |
| HUS8985 | *HUS1435[Pvha-6::LET-413::GFP]; HUS5033 [Phsp16.2::Cas9::tbb-2 3’+PU6-SMAP-1::sgRNA I&II&III, Podr-1::gfp]* | Genetic cross | This study |
| HUS8976 | *Pvha-6::ARF-1.2::GFP; Phsp16.2::Cas9::tbb-2 3’+PU6-SMAP-1::sgRNA I&II&III, Podr-1::gfp; Pvha-6::SMAP-1(R60A)::mCherry* | Microinjection | This study |
| HUS5368 | *HUS4381[Pvha-6::ARF-1.2::GFP]; HUS5252[Pvha-6-SMAP-1::mCherry]* | Genetic cross | This study |
| HUS8963 | *Pvha-6::COPG-1::GFP; Pvha-6::mCherry::P4M* | Microinjection | This study |
| HUS8966 | *Pvha-6::COPB-1::GFP; Pvha-6::mCherry::P4M* | Microinjection | This study |
| HUS8968 | *Pvha-6::APT-9::GFP; Pvha-6::mCherry::P4M* | Microinjection | This study |
| HUS8974 | *Pvha-6::APT-9::GFP* | Microinjection | This study |
| HUS8984 | *HUS8974[Pvha-6::APT-9::GFP]; HUS5033 [Phsp16.2::Cas9::tbb-2 3’+PU6-SMAP-1::sgRNA I&II&III, Podr-1::gfp]* | Genetic cross | This study |
